# Downregulation of *SIRT1* and *GADD45G* genes and left atrial fibrosis induced by right ventricular-dependent pacing in a complete atrioventricular block pig model

**DOI:** 10.17305/bb.2023.9636

**Published:** 2024-04-01

**Authors:** Wei-Chieh Lee, Yu-Sheng Lin, Man-Jing Chen, Wan-Chun Ho, Huang-Chung Chen, Tzu-Hao Chang, Ping-Yen Liu, Mien-Cheng Chen

**Affiliations:** 1 Institute of Clinical Medicine, College of Medicine, National Cheng Kung University, Tainan, Taiwan; 2 Department of Cardiology, Chi Mei Medical Center, Tainan; School of Medicine, College of Medicine, National Sun Yat-sen University, Kaohsiung, Taiwan; 3 Division of Cardiology, Department of Internal Medicine, Kaohsiung Chang Gung Memorial Hospital, Chang Gung University College of Medicine, Kaohsiung, Taiwan; 4 Division of Cardiology, Chang Gung Memorial Hospital, Chiayi, Taiwan; 5 Graduate Institute of Biomedical Informatics, Taipei Medical University, Taipei, Taiwan; 6 Division of Cardiology, Department of Internal Medicine, National Cheng Kung University Hospital, College of Medicine, National Cheng Kung University, Tainan, Taiwan

**Keywords:** Atrial fibrosis, atrioventricular block (AVB), sirtuin signaling pathway, SIRT1, right ventricular (RV) pacing

## Abstract

The molecular and genetic mechanisms underlying left atrial (LA) enlargement and atrial fibrosis following right ventricular (RV)-dependent pacing remain unclear. Our objective was to investigate genetic expressions in the LA of pigs subjected to RV pacing for atrioventricular block (AVB), as well as to identify the differential gene expressions affected by biventricular (BiV) pacing. We established an AVB pig model and divided the subjects into three groups: a sham control group, an RV pacing group, and a BiV pacing group. Differential expression genes (DEGs) analyses conducted through next-generation sequencing (NGS) and enrichment analyses were employed to identify genes with altered expression in the LA myocardium. The RV pacing group showed a significant increase in extracellular fibrosis in the LA myocardium compared to the control group. NGS analysis revealed suppressed expression of the sirtuin signaling pathway in the RV pacing group. Among the DEGs within this pathway, *GADD45G* was found to be downregulated in the RV pacing group and upregulated in the BiV pacing group. Remarkably, the BiV pacing group exhibited elevated levels of GADD45G protein. In our study, we observed significant downregulation of *SIRT1* and *GADD45G* genes, which are associated with the sirtuin signaling pathway, in the LA myocardium of the RV pacing group when compared to the control group. Moreover, these genes, which were downregulated in the RV pacing group, displayed a noteworthy upregulation in the BiV pacing group when compared to the RV pacing group.

## Introduction

In an aging population characterized by a growing prevalence of symptomatic bradycardia, including conditions, such as sick sinus syndrome and complete AVB (CAVB), the global demand for permanent pacemakers (PPMs) has escalated. This increase aims to mitigate cardiac morbidity and mortality linked to symptomatic bradycardia [1, 2]. Nonetheless, chronic use of right ventricular (RV) pacing has been shown to have adverse effects on left ventricular (LV) performance, potentially resulting in left atrial (LA) enlargement due to atrioventricular (AV) dyssynchrony [3–6]. The mechanisms driving LA enlargement in long-term RV pacing patients have been elucidated, particularly in cases of pacemaker syndrome. These mechanisms include hemodynamic deterioration due to AV synchrony loss, mitral regurgitation, inter- or intra-ventricular dyssynchrony, arrhythmia induction, and activation of neuroendocrine reflexes [7]. Observations have indicated that LA enlargement occurs subsequent to RV pacing for CAVB [5], accompanied by a concomitant rise in the incidence of atrial fibrillation (AF) linked to LA enlargement [8]. In various populations, such as healthy individuals [9], elderly adults [10], patients with nonrheumatic heart disease [11], and those who have undergone mitral valve surgery [12], a larger atrial size has been correlated with an increased likelihood of developing AF. In patients with CAVB and RV pacing, the incidence of induced AF is 11.9% [13], surpassing the rate observed in the general population [14]. Notably, the incidence of LA enlargement significantly influences prognosis across diverse populations, particularly among high-risk individuals such as those with heart failure and stroke [15]. For patients with heart failure, biventricular (BiV) pacing has demonstrated a marked reduction in LA volume after synchrony compared to non-BiV pacing approaches [16, 17]. It is imperative to delve into the molecular mechanisms that drive LA enlargement in individuals with CAVB and RV pacing. By doing so, we aim to prevent the development of RV pacing-induced LA enlargement and subsequent AF.

The molecular and genetic mechanisms that drive LA enlargement and structural remodeling following RV pacing have yet to be fully elucidated. This study was conducted with the intent of unraveling the distinct molecular and genetic expressions within the LA myocardium resulting from RV pacing, as well as the alterations or reversals of these expressions facilitated by BiV pacing in the CAVB pig model. To achieve this, we conducted an enrichment analysis of the differentially expressed genes (DEGs) using Ingenuity Pathway Analysis. The primary aim was to pinpoint potential pathways that contribute to the pathological changes observed in the LA myocardium following RV pacing.

## Materials and methods

### Study animals and the creation of RV septal-dependent pacing model and BiV pacing model

**Figure S1.**
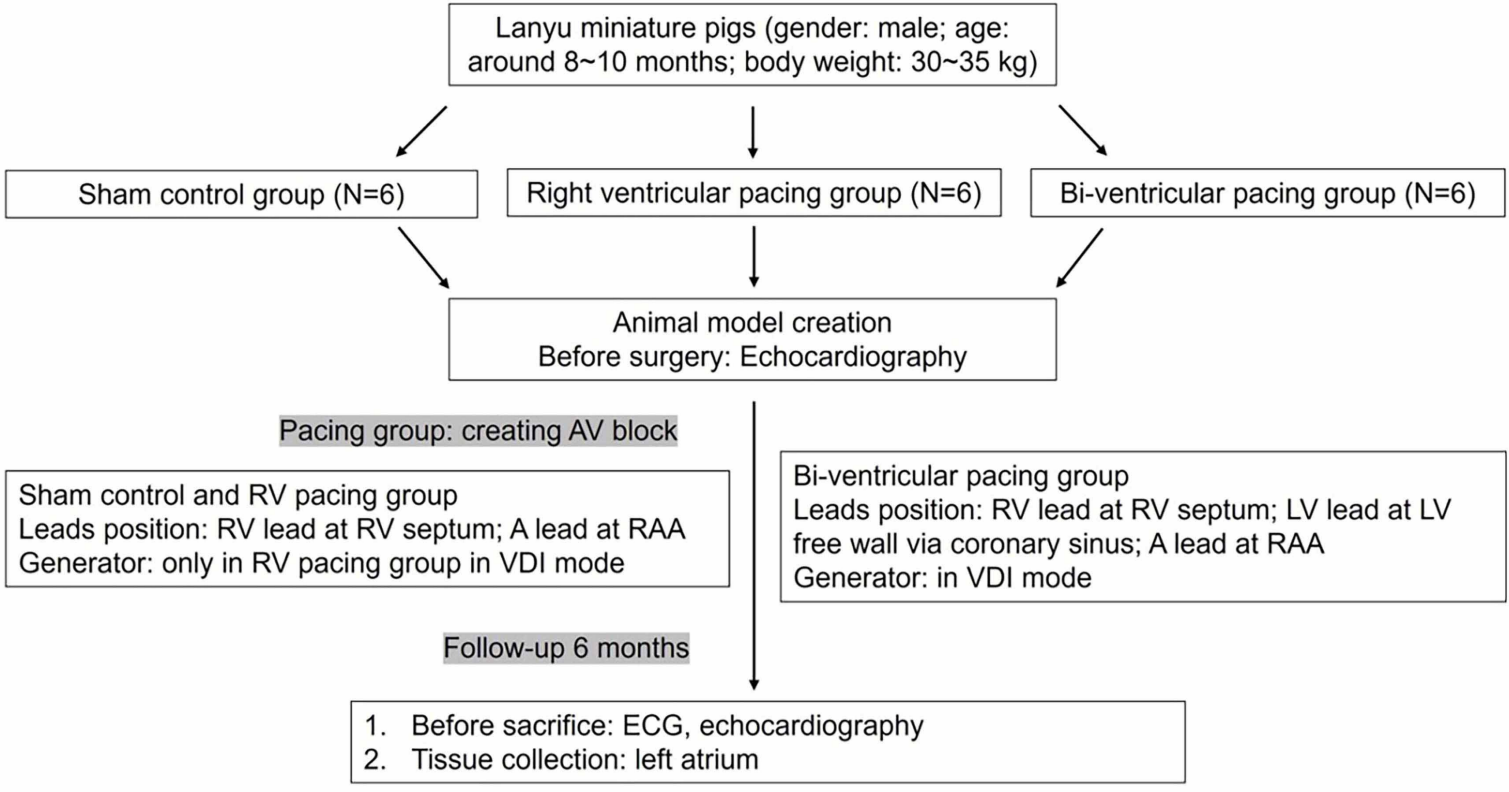
**Grouping algorithm**. AV: Atrioventricular; RV: Right ventricle; A: Atrial; RAA: Right atrial appendage; VDI: Ventricular pacing, dual sensing with inhibition; LV: Left ventricle; ECG: Electrocardiography.

The study included 18 male Lanyu miniature pigs, divided into three distinct groups: the sham control group (*N* ═ 6), the RV pacing group (*N* ═ 6), and the BiV pacing group (*N* ═ 6) (Figure S1). Initially, the BiV pacing was subjected to RV pacing over a period of three months, subsequently followed by BiV pacing for the next three months. All interventions and echocardiographic measurements were performed under anesthesia. Each pig underwent a surgical procedure to establish a pacemaker-dependent model at an average age of 8–10 months. Following the surgery, the pigs were monitored for a period of six months before being euthanized for analysis.

### Implantation of pacemaker and leads and creation of an atrioventricular block

Prior to the surgical procedure, all pigs underwent general anesthesia administered through endotracheal intubation with 2.0%–4.0% isoflurane. Intramuscular premedication involved atropine (1 mg/kg), ketamine (12 mg/kg), and xylazine (1.2 mg/kg). Following an intravenous infusion of 500 mg cefazolin via the ear vein, the procedure for generator and lead implantation was initiated. A pocket approximately 4 cm in length was created over the left paratracheal region. Pacing leads were introduced through the external jugular vein using a cut-down method. With fluoroscopic guidance, an RV screw-in bipolar lead (Abbott, St. Paul, MN, USA) was inserted into the RV and affixed to the septum. The final RV lead position adhered to two criteria: (1) a threshold below 1.0 V with a pulse width of 0.4 ms and an R-wave amplitude exceeding 4.0 mV; and (2) positive electric axes in inferior leads (II, III, and aVF). Subsequently, a right atrial screw-in bipolar lead (Abbott, St. Paul, MN, USA) was placed in the right atrium (RA) and secured at the RA appendage under fluoroscopic guidance. The threshold was verified to be below 1.0 V at a pulse width of 0.4 ms, and the P-wave amplitude was higher than 1.0 mV. In the BiV pacing group, a LV lead was introduced through the coronary sinus and positioned on the LV lateral wall under fluoroscopic guidance. The placement was confirmed with a threshold below 1.5 V at a pulse width of 0.5 ms. Once the leads’ extravenous portions were secured over the paratracheal muscle and connected to the generator, 1.0 g of vancomycin powder was dispersed within the pacemaker pocket. The generator was configured in the ventricular pacing and dual-sensing (VDD) mode, synchronized to the physiological sinus rate of the pigs. The output setting was twice the threshold, and the sensitivity setting was set at 0.5 times the P or R wave. After RV lead placement, atrioventricular nodal ablation was performed using a thermal control ablation catheter guided by fluoroscopy and electrophysiology. Ablation settings were established at 50 W and 60 ^∘^C until CAVB was achieved. This method had been previously validated in our research [18]. Both RV pacing and BiV pacing were fully dependent, ensuring 100% pacing.

### Transthoracic echocardiography

All echocardiographic procedures were conducted using a GE Echocardiographic System (Vivid7; GE-Vingmed, Horten, Norway). Echocardiography was carried out both prior to pacemaker implantation and during the 6-month follow-up period. Following intramuscular administration of atropine (1 mg/kg), ketamine (12 mg/kg), and xylazine (1.2 mg/kg) for anesthesia, the echocardiographic assessments were conducted. During this process, parameters, such as LA area, LA volume, and LA ejection fraction (LAEF), were measured and recorded.

### Specimen storage

After euthanasia, which was performed under general anesthesia, the LA tissues’ lateral wall and septal tissues were procured from the pigs. A portion of these atrial tissues was promptly immersed in liquid nitrogen at −80 ^∘^C for subsequent RNA analyses. Furthermore, some atrial tissues were placed within a tissue Tek^®^ container, subsequently filled with tissue Tek^®^ optimum cutting temperature compound (Sakura^®^ Finetek, CA, USA), and then subjected to freezing in liquid nitrogen for future histochemical assessments. Additionally, a subset of the tissues was promptly fixed in 3.7% buffered formalin and subsequently embedded in paraffin for histological examination.

### Masson’s trichrome staining

Sections of the LA myocardium were subjected to staining and subsequent examination utilizing a modified Masson’s trichrome stain kit (ScyTek Laboratories, Inc., Logan, UT, USA) in accordance with the manufacturer’s guidelines. Briefly, 5-µm sections were deparaffinized, fixed using Bouin’s solution, and stained using Weigert’s iron hematoxylin solution. This was followed by incubation in Biebrich scarlet/acid fuchsin solution that was contained within a phosphomolybdic/phosphotungstic acid solution, and then further incubation in aniline blue and acetic acid. After dehydration, the sections were mounted and visualized using an Olympus DP70 microscope. The extent of fibrosis, as indicated by the percentage of positively stained area, was calculated employing Image Pro Plus software (version 6.0; Media Cybernetics, Silver Spring, MD, USA).

### Western blotting

Protein extracts from LA tissues were prepared using CelLytic™ MT Cell Lysis Reagent (Sigma-Aldrich, St. Louis, MO, USA). The resulting homogenates were centrifuged at 14,000 rpm for 30 min at 4 ^∘^C to yield supernatants. The protein concentrations of the samples were determined using the Bradford method (Bio-Rad Inc., Hercules, CA, USA) following the manufacturer’s guidelines. For electrophoresis, protein extracts (30 µg) were separated on 10%–15% acrylamide SDS-PAGE gel at room temperature for 1 h and then electrotransferred onto PVDF membranes for 1.5 h on ice. Subsequently, the membranes were blocked at room temperature for 1 h using Tris-buffered saline containing 0.1% Tween-20 (TBST) and either 5% (w/v) nonfat dry milk or 2% (w/v) bovine serum albumin. Primary antibodies, including cleaved caspase-3 (dilution of 1:1000, Abcam 13847, Cambridge, UK), TNFR1 (dilution of 1:5000, Cell Signaling Technology 3736, Beverly, MA, USA), TGF-β (dilution of 1:1000, Abcam 31013, Cambridge, UK), and collagen I (dilution of 1:1000, Abcam 6308, Cambridge, UK), were applied to the blots and allowed to react at 4 ^∘^C overnight in 5% nonfat dry milk or 2% bovine serum albumin. After washing the blots three times with TBST, they were incubated at room temperature for 1 h with a horseradish peroxidase-labeled secondary antibody (dilution of 1:5000) in TBST containing either 5% nonfat dry milk or 2% bovine serum albumin. Subsequent to another set of three washes, the blots were treated with Immobilon Western chemiluminescent HRP substrate (Millipore, Burlington, MA, USA). All values specific to the evaluated proteins were normalized to α-sarcomeric actin (dilution of 1:5000, Sigma Aldrich 2172, St. Louis, MO, USA). The resulting chemiluminescence was quantified using a BioSpectrum 810 imaging system (UVP) (Analytik Jena, Germany).

### NGS and quantitative determination of RNA of the LA myocardium

#### RNA extraction

RNA extraction from atrial tissue was carried out using a RiboPureTM kit (Ambion, Grand Island, NY, USA) following the manufacturer’s instructions. The quality of the extracted RNA was evaluated using an Agilent 2100 Bioanalyzer (Agilent Technologies, Santa Clara, CA, USA). Only samples meeting the criteria of an optical density ratio (260/280) greater than 1.8 and an RNA integrity number exceeding 7.0 were chosen for subsequent steps, including NGS.

#### Library preparation for transcriptome sequencing

For RNA sample preparation, 1 µg of RNA per sample was utilized as input. The TruSeq stranded mRNA library prep Kit (cat# RS-122-2101, Illumina, San Diego, CA, USA) was employed to generate sequencing libraries following the manufacturer’s guidelines. Index codes were incorporated to assign sequences to individual samples. In summary, the procedure involved purifying mRNA from total RNA utilizing poly T oligo-attached magnetic beads, followed by fragmentation through heating. First-strand cDNA synthesis was carried out using SuperScript II reverse transcriptase. Subsequent PCR amplification utilized 2X PCR Master Mix. After the 3’ ends of DNA fragments were adenylated, adaptors were ligated, and library fragments were purified using the AMPure XP system (Beckman Coulter, Beverly, USA). The quality of the final libraries was evaluated using an Agilent Bioanalyzer 2100 system with DNA high-sensitivity chips. Sequencing was performed on an Illumina NovaSeq6000 platform, generating 150 bp paired-end reads.

#### Bioinformatics analysis

The Trimmomatic program was employed to eliminate low-quality bases and sequencing adapters from the raw data generated by the Illumina sequencer. Subsequently, the reads were aligned to reference transcript sequences using the Bowtie2 software. The gene expression levels were calculated utilizing RSEM with maximum-likelihood abundance estimates achieved through the expectation–maximization algorithm. DEGs between two groups were defined as genes exhibiting expression differences with a log2 fold change (FC) greater than 1.5 and *P* values lower than 0.05. For the analysis of canonical pathway networks, Ingenuity Pathway Analysis was utilized. Activation z-score analysis was conducted to gauge the activation states (increased or decreased) of pathways influenced by DEGs. In this context, a z-score above 0 indicates a significant increase in predictions for a biological function compared to decreases, and vice versa (z-score below 0).

#### Quantitative determination of RNAs by real-time PCR

RNA samples were quantified using a spectrophotometer. First-strand cDNAs were synthesized using reverse transcriptase and oligo (dT) primers. Real-time quantitative PCR was performed using an ABI Prism 7500 FAST sequence detection system (Applied Biosystems, CA, USA) and SYBR Green PCR Master Mix (Qiagen, CA, USA). The results were normalized to *GAPDH* gene expression (endogenous control).

### Ethical statement

All animals received appropriate care as stipulated by the Guide for the Care and Use of Laboratory Animals published by Taiwan’s National Institutes of Health. The study complied with the Declaration of Helsinki and the animal procedures were approved by the Institutional Animal Care and Committee (IACUC) at Chang Gung Memorial Hospital (IACUC Number: 2019121803).

### Statistical analysis

The data is reported as the mean ± standard error of the mean (SEM). To compare the pacing group with the sham control group, the Mann–Whitney *U* test and one-way analysis of variance were employed. For comparisons of two repeated measurements within either the pacing group or the sham control group, the Wilcoxon signed-rank test was used. The statistical analyses were executed using commercial statistical software (IBM SPSS Statistics 22). A significance level was set at a *P* value of less than 0.05.

## Results

### The changes in LA area and volume

**Figure 1.**
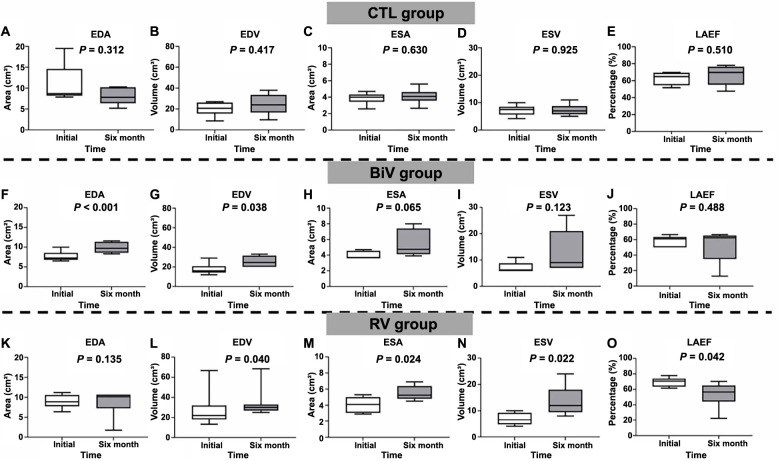
**The area, volume, and performance of LA by transthoracic echocardiography between at PPM implantation and at 6-month follow-up among the 3 groups.** (A–E) In the control group, there was no significant change in the LA EDA, LA EDV, LA ESA, LA ESV, and LA LAEF before PPM implantation and at 6-month follow-up after PPM implantation; (F–J) In the BiV pacing group, the LA EDA (*P* < 0.001) and EDV (*P* ═ 0.038) significantly increased at 6-month follow-up compared with the baseline. There was no significant change in the LA ESA, LA ESV, and LAEF between before PPM implantation and at 6-month follow-up after PPM implantation; (K–O) In the RV group, there was no significant change in the LA ESA before PPM implantation and at 6-month follow-up after PPM implantation. The LA EDV (*P* ═ 0.040), LA ESA (*P* ═ 0.024), and LA ESV (*P* ═ 0.022) significantly increased at 6-month follow-up after PPM implantation compared with baseline. The LAEF significantly decreased at 6-month follow-up compared with the baseline (*P* ═ 0.042). CTL: Control; RV: Right ventricle; BiV: Biventricular; EDA: End-diastolic area; EDV: End-diastolic volume; ESA: End-systolic area; ESV: End-systolic volume; LAEF: Left atrial ejection fraction; PPM: Permanent pacemaker; LA: Left atrium; BiV: Biventricular.

The changes in LA area and volume between values recorded prior to PPM implantation and at the 6-month follow-up across the three groups are illustrated in Figure 1, with group comparisons presented in Table S1. No significant differences were observed among the three groups. In the control group, no substantial alterations were noted in LA area, volume, or LAEF between the baseline and the 6-month follow-up post-PPM implantation. However, in the RV pacing group, significant increases were observed in LA end-diastolic volume (EDV) (Figure 1L; *P* ═ 0.040), LA end-systolic area (ESA) (Figure 1M; *P* ═ 0.024), and LA end-systolic volume (ESV) (Figure 1N; *P* ═ 0.022) at the 6-month follow-up compared to the pre-PPM implantation values. Concurrently, LAEF showed a significant decrease at the 6-month follow-up within the RV group (Figure 1O; *P* ═ 0.042). In the BiV pacing group, the LA end-diastolic area (EDA) (Figure 1F; *P* < 0.001) and LA EDV (Figure 1G; *P* ═ 0.038) exhibited significant increments at the 6-month follow-up in comparison to the baseline. However, no disparity was observed in LAEF (Figure 1J; *P* ═ 0.448) between the period before PPM implantation and the 6-month follow-up in the BiV pacing group.

### Atrial fibrosis developed in the LA myocardium following pacing

**Figure 2.**
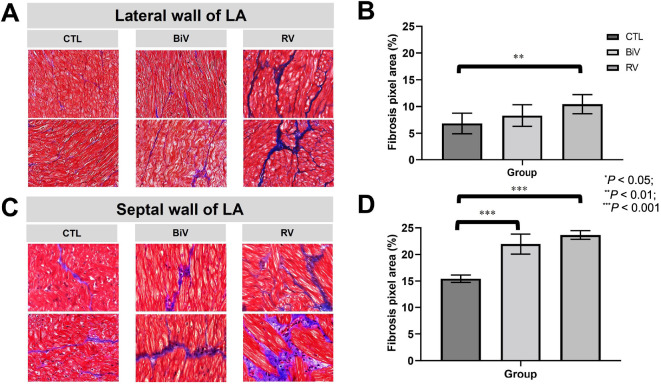
**Masson’s trichrome staining of LA lateral wall and septum**. (A and B) The area of extracellular fibrosis was significantly greater in the RV pacing group than in the sham control group at the LA lateral wall. The area of extracellular fibrosis did not differ between the RV pacing group and the BiV pacing group at the LA lateral wall; (C and D) The area of extracellular fibrosis was significantly greater in the RV pacing group and the BiV pacing group than in the sham control group at the LA septum. The area of extracellular fibrosis did not differ between the RV pacing group and the BiV pacing group at the LA septum. *P < 0.05; **P < 0.01; ***P < 0.001. LA: Left atrium; CTL: Control; BiV: Biventricular; RV: Right ventricle.

The extent of extracellular fibrosis in the mid-myocardial layer of the LA myocardium was notably greater in the RV pacing group in comparison to the sham control group, both in the LA lateral wall (Figure 2A and 2B) and the LA septum (Figure 2C and 2D). Interestingly, this difference between the groups was more pronounced in the LA septum as opposed to the LA lateral wall. Furthermore, there was no discernible distinction in the extent of extracellular fibrosis in the mid-myocardial layer of the LA myocardium when comparing the RV pacing group to the BiV pacing group. Therefore, the fibrosis was not completely reversed in our animal model even after three months of BiV pacing subsequent to three months of RV pacing.

**Figure 3.**
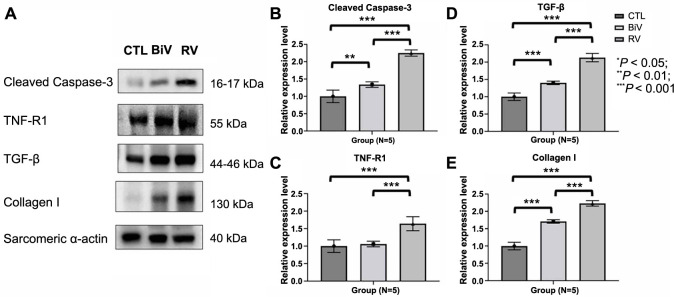
**Immunoblotting study of cleaved casapase-3, TNFR1, TGF-β, and collagen I of LA myocardium.** (A) Immunoblotting study of LA myocardium for apoptosis and fibrosis markers. Immunoblotting study showed that the expressions of cleaved casapase-3 (B), TNFR1 (C), TGF-β (D), and collagen I (E) were significantly increased in the RV pacing group compared to control and BiV pacing groups. Moreover, the expressions of cleaved casapase-3 (B) and collagen I (E) was significantly increased in the BiV pacing group compared to the control group. *P < 0.05; **P < 0.01; ***P < 0.001. CTL: Control; BiV: Biventricular; RV: Right ventricle; TGF-β: Transforming growth factor-beta; TNFR1: Tumor necrosis factor receptor 1; LA: Left atrium.

### Increased expression of fibrotic and apoptosis markers in the LA myocardium following pacing

The expressions of cleaved caspase-3 and tumor necrosis factor receptor 1 (TNFR1) (Figure 3B and 3C), indicative of apoptosis, as well as the expressions of transforming growth factor-β (TGF-β) and collagen I (Figure 3D and 3E), representative of fibrosis, in the LA myocardium, exhibited a notable increase in the RV pacing group compared to the sham control group. Furthermore, both the expressions of cleaved caspase-3 and TNFR1 (Figure 3B and 3C), along with the expressions of TGF-β and collagen I (Figure 3D and 3E) in the LA myocardium, demonstrated a significant elevation in the RV pacing group compared to the BiV pacing group. As a result, we employed NGS and conducted a functional enrichment analysis of the LA myocardium to delve into the molecular pathways and genetic alterations associated with fibrosis induced by RV pacing.

**Figure 4.**
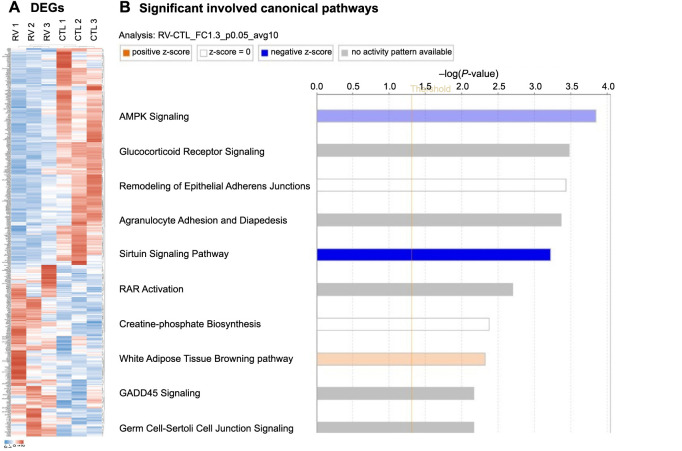
**Functional enrichment analysis of the LA myocardium following 6-month RV pacing compared to the control group**. (A) The heat map graphs for comparison between RV pacing and control groups are depicted. Unsupervised hierarchical clustering of RNA NGS expression sequence tags in the LA myocardium of the RV pacing group and the control group. A total of 387 genes were identified, of which 171 were differentially upregulated and 216 were differentially downregulated. The bar color indicates RNA expression level. Red indicates upregulation; white, no change; blue, downregulation. See Figure S2 for detailed preview of the heatmap. (B) Activation z-score analysis method was used to measure activation states of the canonical pathways affected by DEGs in the LA myocardium. The significant involved canonical pathway of DEGs in the LA myocardium included sirtuin signaling pathway and activation z-score analysis showed inhibition of AMP-activated protein kinase (light blue) and sirtuin signaling pathway (deep blue), and activation of white adipose tissue browning pathway (light orange). CTL: Control; RV: Right ventricle; DEG: Differentially expressed genes; NGS: Next-generation sequencing; LA: Left atrium.

### Functional enrichment analysis: Inhibition of sirtuin signaling pathway in the lateral wall of LA myocardium following six months of RV pacing when compared to sham control

After euthanasia at the 6-month follow-up, dissection of the mid-myocardial layer of the lateral wall of the LA was carried out in both the control and RV pacing groups. A total of 27,250 Sus scrofa genes were employed to identify gene expression profiles within the LA tissues. The gene expression profiles from the lateral wall of LA myocardium derived from three sham control pigs and three RV pacing pigs were utilized for the identification of DEGs through NGS, following six months of RV pacing. The heat map graphs for the comparison between the RV pacing and control groups are depicted in Figure 4A. To elucidate the molecular and genetic mechanisms underlying the impact of RV septal pacing on the gene expression profile within the lateral wall of the LA, we employed Ingenuity Pathway Analysis (Qiagen, Hilden, Germany) for functional enrichment analysis. Furthermore, activation z-score analysis was implemented to assess the activation states (increased or decreased) of the pathways influenced by the DEGs. As illustrated in Figure 4B, the results of the functional analysis of DEGs between the RV pacing and control groups highlighted three pathways: the sirtuin signaling pathway (light blue), AMPK signaling (deep blue), and the white adipose tissue browning pathway (light orange), which were predicted to be significantly inhibited (blue) or activated (orange). Detailed information can be found in Table 1.

**Table 1:** Significant involved canonical pathways and predictive activity derived from DEGs of LA myocardium between RV pacing and control groups using Ingenuity Pathway Analysis

**Comparison**	**Ingenuity canonical pathways**	**−log (*P* value)**	**Ratio**	**z-score**	**Involved genes**
RV pacing vs CTL	Sirtuin signaling pathway	3.22	0.056	−2.673	*ACADL, ATG4D, FOXO1, FOXO3, GABARAPL1, GADD45A, GADD45G, LDHA, NAMPT, NDUFA2, PPARGC1A, RARB, SMARCA5, TIMM50, TUBA4A*
RV pacing vs CTL	AMPK signaling	3.84	0.067	−1.000	*ADRA1B, CAMKK2, CCND1, CHRM2, FOXO1, FOXO3, GNB1L, GYS1, PFKL, PPARGC1A, PPM1E, PRKAG2, PTPA, RAB3A*
RV pacing vs CTL	White adipose tissue browning pathway	2.31	0.064	0.707	*ADCY5, CAMKK2, LDHA, NPPA, NPPB, PPARGC1A, PRKAG2, RARB*

CTL: Control; BiV: Biventricle; RV: Right ventricle; ACADL: Acyl-CoA dehydrogenase long chain; ATG4D: Autophagy-related 4D cysteine peptidase; FOXO1: Forkhead box O1; FOXO3: Forkhead box O3; GABARAPL1: GABA type A receptor-associated protein-like 1; GADD45A: Growth arrest and DNA damage-inducible alpha; GADD45G: Growth arrest and DNA damage-inducible gamma; LDHA: Lactate dehydrogenase A; NAMPT: Nicotinamide phosphoribosyltransferase; NDUFA2: NADH:ubiquinone oxidoreductase subunit A2; PPARGC1A: PPARG coactivator 1 alpha; RARB: Retinoic acid receptor beta; SMARCA5: SWISNF-related, matrix-associated, actin-dependent regulator of chromatin, subfamily a, member 5; TIMM50: Translocase of inner mitochondrial membrane 50; TUBA4A: Tubulin alpha 4a; AMPK: AMP-activated protein kinase; ADRA1B: Adrenoceptor alpha 1B; CAMKK2: Calcium/calmodulin-dependent protein kinase kinase 2; CCND1: Cyclin D1 gene, cholinergic receptor muscarinic 2; GNB1L: Guanine nucleotide-binding protein subunit beta-like protein 1; GYS1: Glycogen synthase 1; PFKL: Phosphofructokinase, liver type; PPM1E: Protein phosphatase, Mg2+/Mn2+ dependent 1E; PRKAG2: Protein kinase AMP-activated non-catalytic subunit Gamma 2; PTPA: Protein phosphatase 2 phosphatase activator; RAB3A: Ras-related protein rab-3A; ADCY5: Adenylyl cyclase 5; NPPA: Natriuretic peptide A; NPPB: Natriuretic peptide B; DEG: Differentially expressed genes; LA: Left atrium.

Remarkably, the sirtuin signaling pathway exhibited the lowest z-score of −2.673, involving genes, such as acyl-CoA dehydrogenase long chain (*ACADL*), autophagy-related 4D cysteine peptidase (*ATG4D*), forkhead box O1 (*FOXO1*), *FOXO3*, GABA type A receptor-associated protein-like 1 (*GABARAPL1*), growth arrest and DNA damage-inducible alpha (*GADD45A*), *GADD45G*, lactate dehydrogenase A (*LDHA*), nicotinamide phosphoribosyltransferase (*NAMPT*), NADH:ubiquinone oxidoreductase subunit A2 (NDUFA2), PPARG coactivator 1 alpha (*PPARGC1A*), retinoic acid receptor beta (*RARB*), SWISNF-related, matrix-associated, actin-dependent regulator of chromatin, subfamily a, member 5 (*SMARCA5*), translocase of the inner mitochondrial membrane 50 (*TIMM50*), and tubulin alpha 4a (*TUBA4A*) (as detailed in Table 1). Among these, three genes (*ATG4D*, *TIMM50*, and *TUBA4A*) were predicted to increase the activation of the sirtuin signaling pathway (as outlined in Table 2), while 12 genes (*ACADL, FOXO1, FOXO3, GABARAPL1, GADD45A, GADD45G, LDHA, NAMPT, NDUFA2, PPARGC1A, RARB,* and *SMARCA5*) were predicted to decrease its activation.

**Table 2:** DEGs between RV pacing and control groups in sirtuin signaling pathway

**Symbol**	**Entrez gene name**	**log2 FC(RV pacing/CTL)**	**Predicted to increase or decrease pathway activation**	**Location**	**Type(s)**
*ACADL*	Acyl-CoA dehydrogenase long chain	−0.723	Decrease	Cytoplasm	Enzyme
*ATG4D*	Autophagy-related 4D cysteine peptidase	0.483	Increase	Cytoplasm	Peptidase
*FOXO1*	Forkhead box O1	−0.759	Decrease	Nucleus	Transcription regulator
*FOXO3*	Forkhead box O3	−0.733	Decrease	Nucleus	Transcription regulator
*GABARAPL1*	GABA type A receptor associated protein-like 1	−0.563	Decrease	Cytoplasm	Other
*GADD45A*	Growth arrest and DNA damage inducible alpha	−0.870	Decrease	Nucleus	Other
*GADD45G*	Growth arrest and DNA damage inducible gamma	−1.438	Decrease	Nucleus	Other
*LDHA*	Lactate dehydrogenase A	1.058	Decrease	Cytoplasm	Enzyme
*NAMPT*	Nicotinamide phosphoribosyl transferase	−0.729	Decrease	Extracellular space	Cytokine
*NDUFA2*	NADH:ubiquinone oxidoreductase subunit A2	0.471	Decrease	Cytoplasm	Enzyme
*PPARGC1A*	PPARG coactivator 1 alpha	−1.503	Decrease	Nucleus	Transcription regulator
*RARB*	Retinoic acid receptor beta	−0.539	Decrease	Nucleus	Ligand-dependent nuclear receptor
*SMARCA5*	SWISNF related, matrix associated, actin dependent regulator of chromatin, subfamily a, member 5	−0.435	Decrease	Nucleus	Transcription regulator
*TIMM50*	Translocase of inner mitochondrial membrane 50	0.522	Increase	Cytoplasm	Phosphatase
*TUBA4A*	Tubulin alpha 4a	0.989	Increase	Cytoplasm	Other

DEG: Differentially expressed genes; FC: Fold change; RV: Right ventricle; CTL: Control.

Between the RV pacing group and the control group, the DEGs suggested inhibition of the AMP-activated protein kinase pathway, as indicated by the activation z-score analysis (−log [*P* value]: 3.84; ratio: 0.067; z-score: −1.000). Similarly, the white adipose tissue browning pathway was projected to be stimulated by the DEGs, with an activation z-score analysis (−log [*P* value]: 2.31; ratio: 0.064; z-score: 0.707). This information is further detailed in Table 1 and Figure 4B.

### Comparison of the expression of 15 DEGs in the sirtuin signaling pathway among the three groups

In the comparison among the three groups, the expression of the *GADD45G* gene exhibited downregulation (Log2 FC): −1.438; *P* < 0.001), while the expression of the *TUBA4A* gene displayed upregulation (FC: 1.656; *P* < 0.001) in the LA myocardium of the RV pacing group when compared to the control group. Conversely, in the LA myocardium of the BiV pacing group compared to the RV pacing group, the expression of the *GADD45G* gene showcased upregulation (FC: 0.988; *P* < 0.001), whereas the expression of the *TUBA4A* gene demonstrated downregulation (FC: −0.715; *P* ═ 0.002). Detailed information is presented in Table 3.

**Table 3:** Comparison of the expression of DEGs in sirtuin signaling pathway among the three groups

**Expression pattern between groups**	**Comparison of groups**	**Symbol**	**Entrez gene name**	**Log2FC**	***P* value**
Different expression between RV and BiV	RV pacing vs CTL	*GADD45G*	Growth arrest and DNA damage inducible gamma	−1.438	<0.001
	BiV pacing vs RV pacing			1.656	<0.001
	RV pacing vs CTL	*TUBA4A*	Tubulin alpha 4a	0.988	<0.001
	BiV pacing v RV pacing			−0.715	0.002
Only expression in RV	RV pacing vs CTL	*ATG4D*	Autophagy-related 4D cysteine peptidase	0.483	0.028
	RV pacing vs CTL	*NDUFA2*	NADH:ubiquinone oxidoreductase subunit A2	0.471	0.038
	RV pacing vs CTL	*TIMM50*	Translocase of inner mitochondrial membrane 50	0.522	0.020
	RV pacing vs CTL	*ACADL*	Acyl-CoA dehydrogenase long chain	−0.723	0.022
	RV pacing vs CTL	*SMARCA5*	SWISNF related, matrix associated, actin dependent regulator of chromatin, subfamily a, member 5	−0.435	0.033
Same expression in both RV and BiV	RV pacing vs CTL	*LDHA*	Lactate dehydrogenase A	1.057	<0.001
	BiV pacing vs CTL			0.861	<0.001
	RV pacing vs CTL	*FOXO1*	Forkhead box O1	−0.759	<0.001
	BiV pacing vs CTL			−1.034	<0.001
	RV pacing vs CTL	*FOXO3*	Forkhead box O3	−0.448	<0.001
	BiV pacing vs CTL			−0.180	<0.001
	RV pacing vs CTL	*GABARAPL1*	GABA type A receptor associated protein-like 1	−0.563	0.021
	BiV pacing vs CTL			−0.681	<0.001
	RV pacing vs CTL	*GADD45A*	Growth arrest and DNA damage inducible alpha	−0.870	<0.001
	BiV pacing vs CTL			−0.773	<0.001
	RV pacing vs CTL	*NAMPT*	Nicotinamide phosphoribosyltransferase	−0.729	0.004
	BiV pacing vs CTL			−0.766	0.002
	RV pacing vs CTL	*RARB*	Retinoic acid receptor beta	−0.539	0.018
	BiV pacing vs CTL			−0.563	0.006
	RV pacing vs CTL	*PPARGC1A*	PPARG coactivator 1 alpha	−1.503	<0.001
	BiV pacing vs CTL			−1.888	<0.001

DEG: Differentially expressed genes; FC: Fold change; RV: Right ventricular; BiV: Biventricular; CTL: Control.

Among the genes, five (*ACADL, ATG4D, NDUFA2, SMARCA5,* and *TIMM50*) showed differential expression exclusively in the LA myocardium of the RV pacing group when compared to the control group. Among these, the expressions of *ATG4D, NDUFA2*, and *TIMM50* exhibited upregulation, while *ACADL* and *SMARCA5* showed downregulation. On the other hand, eight genes (*LDHA, FOXO1, FOXO3, GABARAPL1, GADD45A, NAMPT, RARB,* and *PPARGC1A*) displayed differential expression in both the LA myocardium of the RV pacing group and the BiV pacing group when compared to the control group. Among these, the expression of *LDHA* demonstrated upregulation, while the expressions of *FOXO1, FOXO3, GABARAPL1, GADD45A, NAMPT, RARB,* and *PPARGC1A* exhibited downregulation.

### Quantitative determination of RNAs by real-time PCR for the 15 DEGs in the sirtuin signaling pathway among the three groups

**Figure 5.**
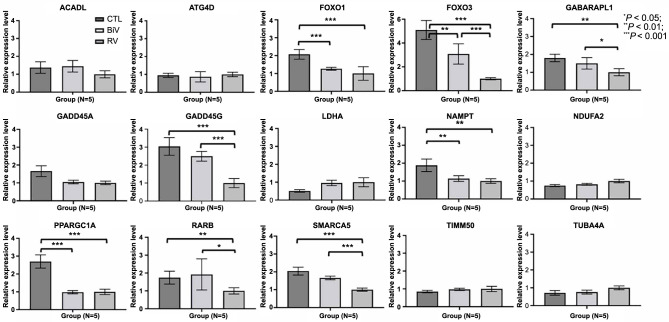
**Quantitative determination of RNAs by real-time polymerase chain reaction for the 15 DEGs in sirtuin signaling pathway among the three groups.** *P< 0.05; **P < 0.01; ***P < 0.001. CTL: Control; BiV: Biventricular; RV: Right ventricle; ACADL: Acyl-CoA dehydrogenase long chain; ATG4D: Autophagy-related 4D cysteine peptidase; FOXO1: Forkhead box O1; FOXO3: Forkhead box O3; GABARAPL1: GABA type A receptor-associated protein-like 1; GADD45A: Growth arrest and DNA damage-inducible alpha; GADD45G: Growth arrest and DNA damage-inducible gamma; LDHA: Lactate dehydrogenase A; NAMPT: Nicotinamide phosphoribosyltransferase; NDUFA2: NADH:ubiquinone oxidoreductase subunit A2; PPARGC1A: PPARG coactivator 1 alpha; RARB: Retinoic acid receptor beta; SMARCA5: SWISNF-related, matrix-associated, actin-dependent regulator of chromatin, subfamily a, member 5; TIMM50: Translocase of inner mitochondrial membrane 50; TUBA4A: Tubulin alpha 4a; DEG: Differentially expressed genes.

Quantitative determination of RNAs via real-time PCR was conducted for six pigs in each group. Details are shown in Figure 5. The expressions of *ACADL, ATG4D, GADD45A, LDHA, NDUFA2, TIMM50,* and *TUBA4A* in the LA myocardium exhibited no significant differences among the three groups. Conversely, in the LA myocardium of the control group, the expressions of *FOXO1, FOXO3, GABARAPL1, GADD45G, NAMPT, PPARGC1A, RARB,* and *SMARCA5* were significantly higher compared to the RV pacing group. Similarly, in the LA myocardium of the control group, the expressions of *FOXO1, FOXO3, NAMPT,* and *PPARGC1A* remained notably higher compared to both the RV pacing and BiV pacing groups. Furthermore, in the LA myocardium of the BiV pacing group, the expressions of *FOXO3, GABARAPL1, GADD45G, RARB,* and *SMARCA5* were significantly higher than those in the RV pacing group.

**Figure 6.**
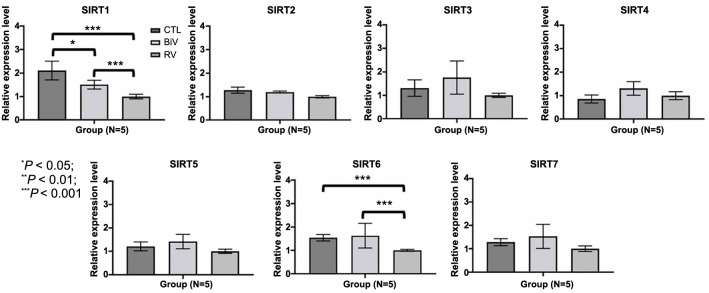
**Quantitative determination of RNAs by real-time polymerase chain reaction for the seven members of sirtuin protein family.** *P < 0.05; **P < 0.01; ***P < 0.001. CTL: Control; BiV: Biventricular; RV: Right ventricle; SIRT: Sirtuin.

Within the sirtuin protein family, which comprises seven members, the expressions of *SIRT1* and *SIRT6* in the LA myocardium, as determined through real-time polymerase PCR of the control group, were significantly higher compared to the RV pacing group (Figure 6). Moreover, in the LA myocardium, the expression of *SIRT1* and *SIRT6* through real-time polymerase PCR of the BiV pacing group was also significantly higher than that in the RV pacing group (Figure 6). However, the expressions of *SIRT2, SIRT3, SIRT4, SIRT5*, and *SIRT7* in the LA myocardium, determined via real-time polymerase PCR, displayed no significant differences among the three groups.

### Decreased expression of SIRT1 and GADD45G protein in the LA myocardium following pacing

**Figure 7.**
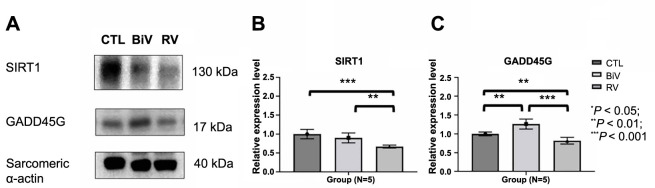
**Immunoblotting study of SIRT1 and GADD45G protein in the LA myocardium following pacing**. (A and B) The levels of SIRT1 protein were significantly lower in the RV pacing group when compared to BiV pacing and control groups. (A and C) The levels of GADD45G protein were significantly lower in the RV pacing group when compared to BiV pacing and control groups. SIRT: Sirtuin; GADD45G: Growth arrest and DNA damage-inducible gamma; LA: Left atrium; BiV: Biventricular; RV: Right ventricle.

The levels of SIRT1 protein were notably lower in the RV pacing group compared to both the BiV pacing and control groups (Figure 7A and 7B). Similarly, the levels of GADD45G protein were significantly lower in the RV pacing group when compared to both the BiV pacing and control groups (Figure 7A and 7C). Remarkably elevated levels of the GADD45G protein were observed in the BiV pacing group.

## Discussion

**Figure 8.**
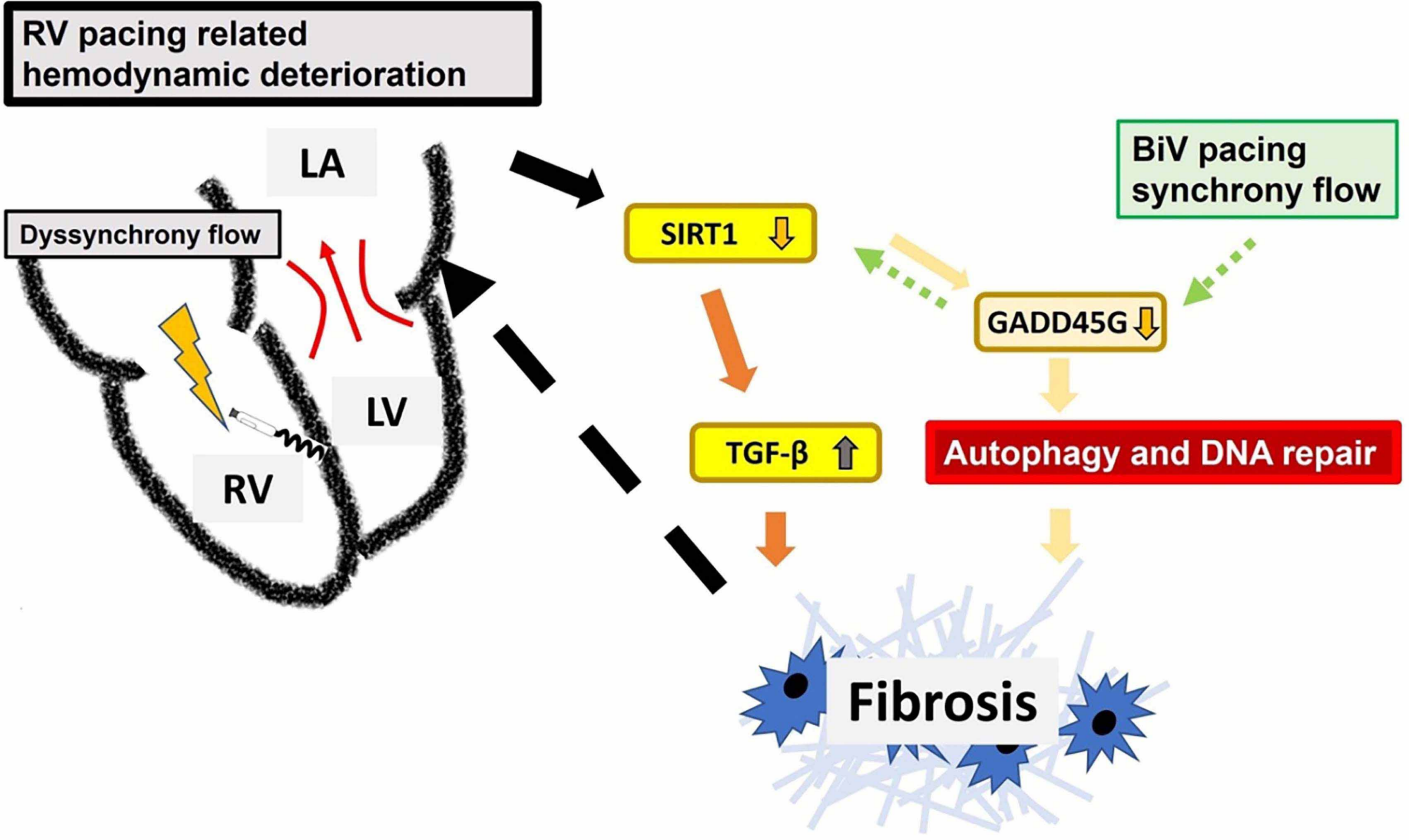
**The proposed mechanism of RV pacing-related LA fibrosis**. After RV pacing, dyssynchrony flow-related hemodynamic deterioration increased oxidative stress, which inhibited the *SIRT1* gene. Inhibition of SIRT1 induced increased expression of TGF-β and fibrosis. The expressions of the *GADD45G* gene were decreased because of the inhibition of SIRT1 and consequently, inhibition of autophagy and DNA repair. BiV pacing may improve pacing related to dyssynchrony flow and increase the expressions of *SIRT1* and *GADD45G* genes in the LA myocardium compared to RV pacing. RV: Right ventricle; SIRT: Sirtuin; GADD45G: Growth arrest and DNA damage-inducible gamma; BiV: Biventricular; TGF-β: Transforming growth factor-beta; LA: Left atrium.

In this study, LA fibrosis and LA enlargement were observed after six months of RV pacing, as evidenced by histopathological changes in the LA myocardium and echocardiographic parameters. The expressions of cleaved caspase-3, TNFR1, TGF-β, and collagen I in the LA myocardium were significantly higher in the RV pacing group compared to the control group. NGS and enrichment analysis utilizing Ingenuity Pathway Analysis between the RV pacing and control groups revealed a significant inhibition of the sirtuin signaling pathway influenced by the DEGs in the LA myocardium. When considering the comparison of DEG expression in the sirtuin signaling pathway and quantitative RNA determination through real-time PCR across the three groups, only the expression of the *GADD45G* gene demonstrated downregulation in the RV pacing group and upregulation in the BiV pacing group. Moreover, the expressions of SIRT1 and GADD45G, both at the gene and protein levels, expressions in the LA myocardium were significantly reduced in the RV pacing group compared to the control group, but notably upregulated in the BiV pacing group relative to the RV pacing group. Consequently, the downregulation of SIRT1 and GADD45G expression might play a pivotal role in the development of LA enlargement and fibrosis following RV-dependent pacing. The proposed mechanism of LA fibrosis related to RV pacing is illustrated in Figure 8. Finally, similar to the RV pacing group, the BiV pacing group did not significantly improve LA EDA and LA EDV as well as atrial fibrosis by histological study with Masson’s trichrome staining at the 6-month follow-up in comparison to the baseline, the expressions of TGF-β and collagen I in the LA myocardium by the more delicate method, i.e., western blotting, exhibited a notable increase in the RV pacing group compared to the sham control group and BiV pacing group. In contrast to the RV pacing group, LA EDA did not significantly increase at the 6-month follow-up in comparison to the baseline in the BiV pacing group; consequently, no disparity was observed in LAEF between the period before PPM implantation and the 6-month follow-up.

Sirtuins comprise a family of nicotine adenine dinucleotide-dependent deacetylating enzymes initially recognized as crucial modulators of lifespan in yeast, Caenorhabditis elegans, Drosophila, and mice [19]. While seven mammalian sirtuins are known, much focus centers on SIRT1, the ortholog of silent information regulator protein 2 pivotal for yeast longevity [20]. SIRT1 functions as a molecular regulator of lifespan, pivotal in maintaining equilibrium between cellular growth and survival. Its substrates encompass proteins that influence chromatin folding, metabolic control, and stress responses, linking SIRT1 to aging, cancer, cardiovascular diseases, and neurodegenerative disorders [21, 22]. SIRT1 activation mitigates cardiac fibrosis in rodent pressure overload models by altering Smad2/3 transactivation [23]. Additionally, SIRT1 overexpression curbs TGF-β excess induction; it is noted that SIRT1 expression is diminished in the LA appendage of AF patients versus sinus rhythm patients [24]. This makes SIRT1 a potential therapeutic target to counteract cardiac and LA fibrosis, crucial factors in AF development [25]. The family of stress-inducible GADD45-like proteins mediates stress-responsive MTK1/MEKK4 MAPKKK activation, prompting p38/JNK activation and apoptosis [26]. SIRT1 activation, in turn, boosts GADD45G upregulation [27]. GADD45 proteins regulate DNA repair, cell cycle control, apoptosis, and genotoxic stress [26]. This suggests that SIRT1 might have a cardioprotective role, influencing DNA repair and oxidative stress, both linked to AF pathogenesis [25]. This study evidenced that SIRT1 and GADD45G genes and protein expressions in the LA myocardium were reduced in the RV pacing group versus the control group but elevated in the BiV pacing group versus the RV pacing group. In the RV group, LA volume notably increased at the 6-month follow-up after PPM implantation compared with baseline. A significantly larger area of extracellular fibrosis was observed in the RV pacing group compared to both the control and BiV pacing groups. Moreover, expressions of cleaved caspase-3, TNFR1, TGF-β, and collagen I were markedly higher in the RV pacing group compared to both the control and BiV pacing groups. These findings underscore the role of downregulated *SIRT1* and *GADD45G* genes within the sirtuin signaling pathway in contributing to LA fibrosis and enlargement following RV pacing for CAVB. Additionally, the expression of the *GADD45G* gene might play a pivotal role in the sirtuin signaling pathway related to LA fibrosis. These insights could prove valuable for future research into the impact of RV pacing on atrial enlargement and fibrosis and provide a possible mechanism for LA enlargement in patients with CAVB who have undergone long-term RV pacing.

### Limitations

First, it is important to note that the DEG analyses were conducted on a subset of three pigs per group, potentially leading to discrepancies when compared to the comprehensive quantitative determination of RNAs using real-time PCR on all pigs across three groups. Second, the design of the BiV pacing group deviates from the RV pacing model for an extended period. As a result, the definitive causal link between the DEGs and the reversal of LA fibrosis via BiV pacing necessitates further investigation. Nevertheless, our study does present a comparison of genetic expressions pertaining to LA enlargement across control, RV pacing, and BiV pacing scenarios. We also explore the potential involvement of the sirtuin signaling pathway, which could impact LA enlargement in the context of long-term RV pacing in clinical practice.

## Conclusion

In the CAVB pig model, substantial LA enlargement was observed after six months of RV pacing. Enrichment analysis of DEGs in the LA myocardium between RV pacing and control groups using NGS revealed the inhibition of the sirtuin signaling pathway due to DEGs. The expressions of *SIRT1* and *GADD45G* genes and protein in the LA myocardium were notably downregulated in the RV pacing group compared to the control group, and these downregulated genes were effectively reversed and upregulated in the BiV pacing group compared to the RV pacing group. Additionally, the expressions of cleaved caspase-3, TNFR1, TGF-β, and collagen I in the LA myocardium exhibited significant increases in the RV pacing group compared to the control group, which were subsequently reversed and decreased in the BiV pacing group compared to the RV pacing group. Further validation of these findings through clinical studies is warranted.

## Acknowledgments

The authors thank Man-Jing Chen and Wan-Chun Ho for experimental management. The authors thank Professor Tzu-Hao Chang for the analyses of NGS and enrichment analysis of DEGs.

## Supplemental data

Detailed preview of Figure 4A is available at the following link: https://drive.google.com/file/d/1tpZal7xN4F-wEJ7qOp84EJHDWMwmeyeg/view?usp=sharing

**Table S1:** Echo parameters of LA between groups

**Group**	**CTL**	**BiV**	**RV**	***P* value**
*Baseline*				
ESA (cm^2^)	3.7 ± 0.8	4.0 ± 0.5	4.1 ± 0.9	0.563
EDA (cm^2^)	9.7 ± 4.1	7.7 ± 1.3	8.9 ± 1.6	0.420
ESV (cm^3^)	6.6 ± 2.2	7.2 ± 2.0	7.1 ± 2.3	0.436
EDV (cm^3^)	20.0 ± 6.6	17.7 ± 5.9	22.3 ± 6.3	0.828
LAEF (%)	64.8 ± 11.9	58.5 ± 7.0	67.5 ± 7.7	0.243
*6-month*				
ESA (cm^2^)	4.3 ± 0.9	5.5 ± 1.7	5.2 ± 1.1	0.199
EDA (cm^2^)	9.0 ± 2.2	9.9 ± 1.4	10.2 ± 0.6	0.738
ESV (cm^3^)	8.1 ± 2.4	13.0 ± 8.2	12.6 ± 6.0	0.225
EDV (cm^3^)	24.2 ± 9.0	25.5 ± 5.5	28.4 ± 3.6	0.473
LAEF (%)	63.2 ± 13.6	52.0 ± 21.2	56.7 ± 17.3	0.486

Data are expressed as mean (standard deviation). CTL: Control; RV: Right ventricle; BiV: Biventricular; EDA: End-diastolic area; EDV: End-diastolic volume; ESA: End-systolic area; ESV: End-systolic volume; LAEF: Left atrium ejection fraction; LA: Left atrium.

## Data Availability

The study data are available from the corresponding author upon reasonable request.

## References

[1] Akerström F, Pachón M, Puchol A, Jiménez-López J, Segovia D, Rodríguez-Padial L (2014). Chronic right ventricular apical pacing: adverse effects and current therapeutic strategies to minimize them. Int J Cardiol.

[2] de Vries LM, Dijk WA, Hooijschuur CAM, Leening MJG, Stricker BHC, van Hemel NM (2017). Utilisation of cardiac pacemakers over a 20-year period: results from a nationwide pacemaker registry. Neth Heart J.

[3] Dreger H, Maethner K, Bondke H, Baumann G, Melzer C (2012). Pacing-induced cardiomyopathy in patients with right ventricular stimulation for >15 years. Europace.

[4] Ghani A, Delnoy PPHM, Ottervanger JP, Ramdat Misier AR, Smit JJJ, Elvan A (2011). Assessment of left ventricular dyssynchrony in pacing-induced left bundle branch block compared with intrinsic left bundle branch block. Europace.

[5] Lin Y-S, Guo GB-F, Chen Y-L, Tsai T-H, Pan K-L, Liu W-H (2011). Atrial enlargement in symptomatic heart block patients with preserved left ventricular function: possibly related to atrioventricular dyssynchrony. Int J Cardiol.

[6] Liu W-H, Chen M-C, Chen Y-L, Guo B-F, Pan K-L, Yang C-H (2008). Right ventricular apical pacing acutely impairs left ventricular function and induces mechanical dyssynchrony in patients with sick sinus syndrome: a real-time three-dimensional echocardiographic study. J Am Soc Echocardiogr.

[7] Nielsen JC, Andersen HR, Thomsen PE, Thuesen L, Mortensen PT, Vesterlund T (1998). Heart failure and echocardiographic changes during long-term follow-up of patients with sick sinus syndrome randomized to single-chamber atrial or ventricular pacing. Circulation.

[8] Sparks PB, Mond HG, Vohra JK, Yapanis AG, Grigg LE, Kalman JM (1999). Mechanical remodeling of the left atrium after loss of atrioventricular synchrony. A long-term study in humans. Circulation.

[9] Zacà V, Galderisi M, Mondillo S, Focardi M, Ballo P, Guerrini F (2007). Left atrial enlargement as a predictor of recurrences in lone paroxysmal atrial fibrillation. Can J Cardiol.

[10] Psaty BM, Manolio TA, Kuller LH, Kronmal RA, Cushman M, Fried LP (1997). Incidence of and risk factors for atrial fibrillation in older adults. Circulation.

[11] Vaziri SM, Larson MG, Benjamin EJ, Levy D (1994). Echocardiographic predictors of nonrheumatic atrial fibrillation. The Framingham heart study. Circulation.

[12] Chen M-C, Chang J-P, Chang H-W, Chen C-J, Yang C-H, Chen Y-H (2005). Clinical determinants of sinus conversion by radiofrequency maze procedure for persistent atrial fibrillation in patients undergoing concomitant mitral valvular surgery. Am J Cardiol.

[13] Yamashita T, Murakawa Y, Ajiki K, Omata M (1997). Incidence of induced atrial fibrillation/flutter in complete atrioventricular block. A concept of ’atrial-malfunctioning’ atrio-hisian block. Circulation.

[14] Kannel WB, Wolf PA, Benjamin EJ, Levy D (1998). Prevalence, incidence, prognosis, and predisposing conditions for atrial fibrillation: population-based estimates. Am J Cardiol.

[15] Hoppe UC, Casares JM, Eiskjaer H, Hagemann A, Cleland JG, Freemantle N (2006). Effect of cardiac resynchronization on the incidence of atrial fibrillation in patients with severe heart failure. Circulation.

[16] Kuperstein R, Goldenberg I, Moss AJ, Solomon S, Bourgoun M, Shah A (2014). Left atrial volume and the benefit of cardiac resynchronization therapy in the MADIT-CRT trial. Circ Heart Fail.

[17] Patel DA, Lavie CJ, Milani RV, Shah S, Gilliland Y (2009). Clinical implications of left atrial enlargement: a review. Ochsner J.

[18] Lin YS, Chang TH, Shi CS, Wang YZ, Ho WC, Huang HD (2019). Liver X receptor/retinoid X receptor pathway plays a regulatory role in pacing-induced cardiomyopathy. J Am Heart Assoc.

[19] Zhang D, Li B, Li B, Tang Y (2020). Regulation of left atrial fibrosis induced by mitral regurgitation by SIRT1. Sci Rep.

[20] Imai S, Armstrong CM, Kaeberlein M, Guarente L (2000). Transcriptional silencing and longevity protein Sir2 is an NAD-dependent histone deacetylase. Nature.

[21] Bindu S, Pillai VB, Gupta MP (2016). Role of sirtuins in regulating pathophysiology of the heart. Trends Endocrinol Metab.

[22] Lagouge M, Argmann C, Gerhart-Hines Z, Meziane H, Lerin C, Daussin F (2006). Resveratrol improves mitochondrial function and protects against metabolic disease by activating SIRT1 and PGC-1alpha. Cell.

[23] Bugyei-Twum A, Ford C, Civitarese R, Seegobin J, Advani SL, Desjardins JF (2018). Sirtuin 1 activation attenuates cardiac fibrosis in a rodent pressure overload model by modifying Smad2/3 transactivation. Cardiovasc Res.

[24] Han L, Tang Y, Li S, Wu Y, Chen X, Wu Q (2020). Protective mechanism of SIRT1 on Hcy-induced atrial fibrosis mediated by TRPC3. J Cell Mol Med.

[25] Treviño-Saldaña N, García-Rivas G (2017). Regulation of sirtuin-mediated protein deacetylation by cardioprotective phytochemicals. Oxid Med Cell Longev.

[26] Tamura RE, de Vasconcellos JF, Sarkar D, Libermann TA, Fisher PB, Zerbini LF (2012). GADD45 proteins: central players in tumorigenesis. Curr Mol Med.

[27] Scuto A, Kirschbaum M, Buettner R, Kujawski M, Cermak JM, Atadja P (2013). SIRT1 activation enhances HDAC inhibition-mediated upregulation of GADD45G by repressing the binding of NF-κB/STAT3 complex to its promoter in malignant lymphoid cells. Cell Death Dis.

